# Polypharmacy and medical intensive care unit (MICU) admission and 10-year all-cause mortality risk among hospitalized patients with and without HIV

**DOI:** 10.1371/journal.pone.0276769

**Published:** 2022-10-27

**Authors:** Kirsha S. Gordon, Kristina Crothers, Adeel A. Butt, E. Jennifer Edelman, Cynthia Gibert, Margaret M. Pisani, Maria Rodriguez-Barradas, Christina Wyatt, Amy C. Justice, Kathleen M. Akgün

**Affiliations:** 1 VA Connecticut Healthcare System, West Haven, CT, United States of America; 2 Yale School of Medicine, New Haven, CT, United States of America; 3 VA Puget Sound Health Care System, Seattle, WA, United States of America; 4 Division of Pulmonary, Critical Care & Sleep Medicine, University of Washington, Seattle, WA, United States of America; 5 Department of Medicine, Weill Cornell Medical College, New York, NY, United States of America; 6 VA Pittsburgh Healthcare System, Pittsburgh, PA, United States of America; 7 Center for Interdisciplinary Research on AIDS, Yale School of Public Health, New Haven, CT, United States of America; 8 George Washington University School of Medicine, Washington, DC, United States of America; 9 Washington DC VA Medical Center, Washington, DC, United States of America; 10 Michael E. DeBakey VA Medical Center and Baylor College of Medicine, Houston, TX, United States of America; 11 Duke University School of Medicine, Durham, NC, United States of America; 12 Yale School of Public Health, New Haven, CT, United States of America; Taipei Medical University, TAIWAN

## Abstract

**Objective:**

Medical intensive care unit (MICU) admissions have been declining in people with HIV infection (PWH), but frequency of outpatient polypharmacy (prescription of ≥5 chronic medications) has increased. Among those hospitalized, we examined whether outpatient polypharmacy is associated with subsequent 1-year MICU admission or 10-year all-cause mortality, and if the association varies by HIV status.

**Design:**

Retrospective cohort study.

**Methods:**

Using a national electronic health record cohort of Veterans in care, we ascertained outpatient polypharmacy during fiscal year (FY) 2009 and followed patients for 1-year MICU admission and 10-year mortality. We assessed associations of any polypharmacy (yes/no and categorized ≤4, 5–7, 8–9, and ≥10 medications) with 1-year MICU admission and 10-year mortality using logistic and Cox regressions, respectively, adjusted for demographics, HIV status, substance use, and severity of illness.

**Results:**

Among 9898 patients (1811 PWH) hospitalized in FY2010, prior outpatient polypharmacy was common (51%). Within 1 year, 1532 (15%) had a MICU admission and within 10 years, 4585 (46%) died. Polypharmacy was associated with increased odds of 1-year MICU admission, in both unadjusted (odds ratio (OR) 1.36 95% CI: (1.22, 1.52)) and adjusted models, aOR (95% CI) = 1.28 (1.14, 1.43) and with 10-year mortality in unadjusted, hazard ratio (HR) (95% CI) = 1.40 (1.32, 1.48), and adjusted models, HR (95% CI) = 1.26 (1.19, 1.34). Increasing levels of polypharmacy demonstrated a dose-response with both outcomes and by HIV status, with a stronger association among PWH.

**Conclusions:**

Among hospitalized patients, prior outpatient polypharmacy was associated with 1-year MICU admission and 10-year all-cause mortality after adjusting for severity of illness in PWH and PWoH.

## Introduction

People with HIV infection (PWH) with access to combination antiretroviral (ARV) treatment are living longer and developing multiple chronic conditions requiring non-ARV medications [1]. Polypharmacy, defined as concurrent use of 5 or more chronic medications [2], is associated with adverse drug events, potentially inappropriate medications (PIMS), falls, cognitive impairments, mortality, and hospitalization [3–6]. Polypharmacy and associated adverse outcomes are a particular problem for PWH, who typically cross the threshold for polypharmacy 10 years earlier than people without HIV (PWoH) [7].

Non-ARV polypharmacy has a dose–response association with increased hospitalization risk in PWH as well as PWoH [5]. Medical intensive care unit (MICU) for hospitalized patients, in particular, is associated with high risk for functional disability and mortality [8–12]. Among hospitalized patients, we asked whether 1) polypharmacy from non-ARV medications is associated with both 1-year MICU admission and 10-year all-cause mortality; 2) if there is a dose-response relationship between medication count and these outcomes; and 3) whether these associations differ by HIV status.

## Materials and methods

### Study sample

We used data from the Veterans Aging Cohort Study (VACS), described elsewhere [13]. Briefly, VACS is a cohort of PWH and PWoH matched 1:2 on age, sex, race/ethnicity, and site of care identified from the United States Veterans Health Administration (VA) electronic health record data. For these analyses baseline was from October 1^st^ 2008 to September 30^th^ 2009 (i.e. fiscal year (FY) 2009), during which outpatient non-ARV polypharmacy was measured. We restricted the sample to patients in care who received at least one non-ARV medication from the VA, and hospitalized in the 12 months period after baseline (i.e., FY2010). Hospitalization was identified from the VA (including fee-based, non-VA hospitalizations) and Center for Medicare and Medicaid Services. There were 75185 patients with at least one outpatient non-ARV medication in FY2009. We excluded patients who had any cancer diagnosis except nonepithelial skin cancer (n = 1874); ambiguous HIV status (e.g., suspected seroconvertors) to make a clear comparison between PWH and PWoH (n = 99); PWH who were not virally suppressed (>400 HIV-1 RNA copies/ml) in the last 6 months of FY2009 (n = 9018), as we sought to study polypharmacy among those on successful ARV therapy; those missing VACS index 2.0 scores (a measurement of severity of illness) (n = 4789); and those who were on more than 15 medications to eliminate highly leveraged observations (n = 534). There were 9898 patients hospitalized in FY2010.

Data were obtained from the VA corporate data warehouse and included demographics, hospitalization and outpatient diagnoses (recorded using International Classification of Diseases, Ninth Revision [ICD-9] codes), laboratory results, and dispensed medications from the Pharmacy Benefits Management (PBM) program. HIV status was based on 042. and V08. ICD-9 codes. The United States (U.S.) Medicare claims data available for Veterans were also included in these analyses and were merged with the VACS data to improve ascertainment of hospitalization. Patients were observed for 12 months after FY2009 (baseline) for MICU admission, and after hospital discharge for 10 years for mortality.

### Primary predictor

We determined receipt of all outpatient preparations (i.e., oral, inhaled, or injectable) of non-ARV medications dispensed through the VA using prescription pharmacy fill/refill data; each component of co-formulated medications was counted separately. We excluded prescriptions classified as diagnostic supplies (e.g., glucose test strips); emollients; eye washes and lubricants; soaps, shampoos and soap-free cleaners; mouthwashes; sun protectants and screens; irrigation solutions; ceruminolytics; deodorants and antiperspirants; and contact lens solutions from analyses. Our analysis was restricted to medications that were filled on a chronic basis, defined as at least 90 consecutive days allowing for a 30-day refill window, consistent with previous definitions [14]. Days of medication receipt were calculated based on prescription information, assuming the prescription was taken as directed. We calculated the mean number of unique non-ARV chronic medications received by each patient during baseline by summing the number of days supplied for each medication and dividing the total by 365 days [5].

We defined polypharmacy as ≥5 concurrent non-ARV medications (yes/no), as commonly defined in the literature [2–6]. In addition, medication count was categorized as ≤4 (reference group), 5–7, 8–9, and ≥10 (i.e., hyper-polypharmacy [15–17]) to assess dose response between chronic medication count and outcomes.

To determine whether non-ARV medication profiles changed substantially over the observation time, we compared the FY2009 medication profile with the medication profile of VACS patients alive in FY2018 who met the criteria of our study sample, without the restriction of hospitalization, to evaluate common outpatient non-ARV medication prescriptions.

### Outcomes and covariates

Our outcomes of interest were the first MICU admission within the 12 months after baseline, ascertained using VA bed section and Medicare revenue center codes (12 and 202), and 10-year all-cause mortality since hospital discharge date (S1 Fig). The index date for MICU admission analysis was the start of observation October 1^st^ 2009 and the index date for 10-year mortality analysis was hospital discharge date. Deaths were identified from the VA Vital Status File, which uses data from the Social Security Death Master File, Medicare Vital Status Files, and VA Beneficiary Identification and Records Locator Subsystem.

Demographic variables included age, race/ethnicity, and sex. Other variables of interest were documented smoking status (current, past, or never; missing smoking status was treated as non-smoking) from VA Health Factors, alcohol and drug related diagnoses based on ICD-9 codes (S1 Table), and severity of illness using the VACS Index 2.0 [18, 19] closest to hospital date within the 12 months prior and up to hospital date. The VACS Index 2.0 is a physiologic score predicting risk of all-cause mortality, initially developed among PWH, and includes age, HIV biomarkers (HIV-1 RNA viral load; CD4 cell count), and non-HIV biomarkers (hemoglobin, hepatitis C; FIB-4 to assess liver fibrosis, estimated glomerular filtration rate to assess kidney function, albumin, body mass index, and white blood cell count). Higher values are associated with a greater risk of mortality in PWH and PWoH.

### Analyses

We conducted descriptive analyses of demographic, clinical characteristics, and polypharmacy by MICU and HIV status. We also looked at characteristics by polypharmacy levels (i.e., medication count categories). We used t-test for continuous variables, or a nonparametric counterpart, Wilcoxon test, for non-normally distributed continuous variables, and Chi-square for categorical variables. A side-by-side comparison of the top ten common chronic non-ARV medications in FY2009 and FY2018 was generated. We employed logistic regression models to assess the relationship between polypharmacy, categories of medication count and 1-year MICU admission, and Cox regressions to assess the association between polypharmacy, categories of medication count and time-to-death from hospital discharge date; adjusted for demographics, HIV status, substance use (i.e., smoking, alcohol or drug related diagnosis), and VACS index 2.0 score (closest to hospital admission date for MICU analysis and closest to discharge date for 10-year mortality analysis). We formally tested for an interaction between HIV and polypharmacy, as HIV may be an effect modifier of polypharmacy and our outcome, by including an interaction term in the model; finally, we ran stratified models by HIV status to identify additional differential associations of polypharmacy for outcomes by HIV status. In a sensitivity analysis using Poisson regression, we estimated relative risk (RR) of 1-year MICU admission. In addition, we used a more advanced model investigating the relationship between medication count and MICU that uses a continuous medication count (not categories). Specifically, we ran a restricted cubic spline regression (with 5 knots) looking at medication count and MICU to see if there were substantial differences in results and to provide a more detailed view of the relationship.

Our study was approved by the IRB of the VA Connecticut Healthcare System and Yale University School of Medicine. Statistical analyses were conducted with SAS, version 9.4.

## Results

Our analytic sample consisted of 9898 (1811 PWH) hospitalized patients during FY2010, 1532 (283 PWH) of whom had a MICU admission. After hospital discharge, 4585 (878 PWH) died within a 10-year period. Patients were predominately male (98%), 50–64 years old (66%), Black (49%), and current smokers (58%). In addition, 51% had any polypharmacy and the median (interquartile range (IQR)) non-ARV medications was 5 (2, 7). The median (IQR) VACS index 2.0 score was 43 (34, 56).

In bivariate analysis, hospitalized patients admitted to MICU were more likely to have polypharmacy prior to admission (57% vs. 50%) and to be in the higher medication count groups when compared to patients without a MICU admission: ≤4 (43% vs. 50%), 5–7 (29% vs. 27%), 8–9 (14% vs. 11%), and ≥10 (14% vs. 12%) medications. The p value for trend for MICU and medication count category was p<0.001. Patients admitted to the MICU were also more likely to be ≥65 years old (27% vs. 19%), to report past smoking (20% vs 17%), had higher severity of illness (i.e., higher median VACS index 2.0 score 49 vs. 42) and higher 10-year mortality (69% vs. 42%). There was no difference in MICU admission by HIV status.

Among those with a MICU admission, PWH compared to PWoH had a higher severity of illness (median VACS index 2.0 score (IQR) = 68 (52, 84) vs. 46 (36, 58)), and higher 10-year mortality (74% vs. 68%) (Table 1). There was no difference in demographic factors by HIV status.

**Table 1:** Descriptive statistics of patient’s baseline characteristics by MICU admission and HIV status.

	no MICU, n = 8366			MICU, n = 1532		
Characteristics	PWoH, n = 6838	PWH, n = 1528	p value	PWoH, n = 1249	PWH, n = 283	p value
**Age** (years) [%]			0.52			0.57
<50	14.38	14.66		8.97	10.25	
50–64	67.20	65.77		63.57	65.02	
≥65	18.43	19.57		27.46	24.73	
**Sex** [%]			0.43			1.00
Female	2.57	2.23		1.28	1.06	
Male	97.43	97.77		98.72	98.94	
**Race** [%]			0.006			0.62
White	40.45	43.98		41.47	42.4	
Black	49.78	45.29		50.84	48.41	
Hispanic/Latino	9.77	10.73		7.69	9.19	
**Smoking** [%]			0.32			0.13
Never	24.64	26.24		25.14	22.97	
Current	58.53	58.05		54.52	60.78	
Past	16.83	15.71		20.34	16.25	
**Alcohol related diagnosis** [%]	20.37	14.73	<0.001	19.30	14.13	0.04
**Drug related diagnosis** [%]	18.31	18.26	0.96	15.37	19.43	0.09
**VACS Index score 2.0**, IQR	39 (32, 49)	60 (48, 72)	<0.001	46 (36, 58)	68 (52, 84)	<0.001
**Polypharmacy** (≥5 meds) [%]	50.41	45.88	0.001	57.25	57.24	1.00
**Polypharmacy categories** [%]			0.003			0.41
≤4 medications	49.59	54.12		42.75	42.76	
5–7 medications	27.11	25.65		28.82	30.04	
8–9 medications	10.92	10.60		13.29	15.55	
≥10 medications	12.37	9.62		15.13	11.66	

MICU–medical intensive care unit; PWoH–people without HIV; PWH–people with HIV; IQR–interquartile range

Baseline characteristics also varied with polypharmacy status. With increasing categories of polypharmacy, patients were older, less likely to currently smoke and had higher VACS 2.0 scores (i.e., higher severity of illness). Those with hyper-polypharmacy were less likely to be female, PWH, or have alcohol or drug related diagnosis (Table 2).

**Table 2:** Descriptive statistics of patient’s baseline characteristics by polypharmacy categories.

	Medication count				
	≤ 4	5–7	8–9	≥10	p value
**Age** (years) [%]					<0.001
<50	16.79	11.85	10.01	8.15	
50–64	64.97	67.48	66.22	70.37	
≥65	18.24	20.66	23.77	21.48	
**Sex** [%]					0.63
Female	2.34	2.49	2.32	1.81	
Male	97.66	97.51	97.68	98.19	
**Race** [%]					<0.001
White	37.86	41.47	47.63	47.90	
Black	51.92	48.72	43.16	44.77	
Hispanic/Latino	10.22	9.81	9.20	7.33	
**HIV-status** [%]					0.002
PWoH	80.55	82.27	81.59	85.19	
PWH	19.45	17.73	18.41	14.81	
**Smoke status** [%]					<0.001
Never	25.18	24.45	25.56	24.20	
Current	59.74	58.34	55.76	52.43	
Past	15.08	17.21	18.68	23.37	
**Alcohol related diagnosis** [%]	21.16	18.73	16.00	15.23	<0.001
**Drug related diagnosis** [%]	19.99	17.24	14.57	14.57	<0.001
**VACS index score 2.0**, IQR	42 (33, 55)	44 (34, 57)	45 (35, 57)	44 (34, 55)	<0.001

PWoH–people without HIV; PWH–people with HIV; IQR–interquartile range

### Polypharmacy pattern comparisons

**Fig 1:**
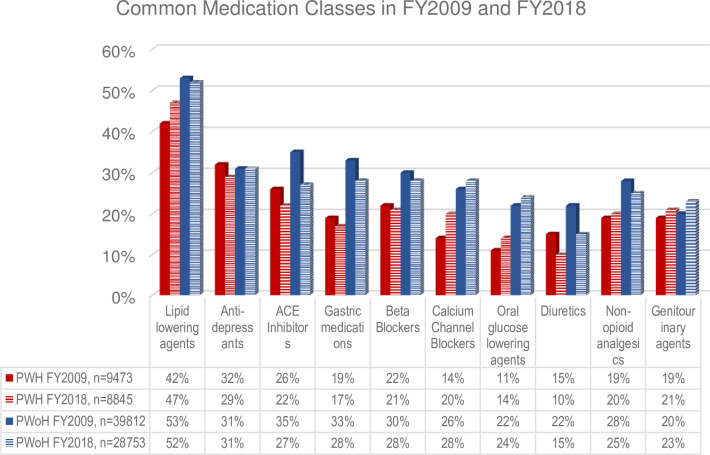
Top ten non-ARV medications in FY2009 and FY2018.

To be able to consider 10-year mortality as an outcome, we had to determine polypharmacy 10 years earlier. In a separate analysis of data from FY 2018 using the same eligibility criteria (PWH = 8845 and PWoH = 28753), we determined whether medication profiles changed. When the FY2009 medication profile was compared to the medication profile of patients in FY2018, the top ten common non-ARV medications were the same (Fig 1). Lipid lowering medications (PWH FY2009 = 42% and FY2018 = 47%; PWoH 53% and 52%) and anti-depressants (PWH FY2009 = 32% and FY2018 = 29%; PWoH = 31% both timeframes) were the most common non-ARVs in both PWH and PWoH. Moreover, 51% of patients had polypharmacy in both FYs, with an increase in FY2018 for hyper-polypharmacy (15% in FY2018 from 12% in FY2009).

### Polypharmacy and 1-year MICU admission

In the unadjusted logistic model, patients with polypharmacy were more likely to have a MICU admission within 12 months, compared to patients without polypharmacy, odds ratio (OR) (95% CI) = 1.36 (1.22, 1.52), p<0.001 (S2 Table). In the unadjusted model using categorical polypharmacy variable, compared to patients who received ≤4 medications, those on 5–7, 8–9, and ≥10 were 28%, 49% and 44% more likely to have a MICU admission, (OR (95% CI) = 1.28 (1.12, 1.45); 1.49 (1.25, 1.77); and 1.44 (1.22, 1.70), respectively; all p<0.01) (Table 3).

**Table 3:** Logistic regression models looking at increments of medication count and MICU admission.

	Unadjusted, n = 9898		Adjusted, n = 9898		PWH, n = 1811		PWoH, n = 8087	
	OR		OR		OR		OR	p value
	(95% CI)	p value	(95% CI)	p value	(95% CI)	p value	(95% CI)	
**5–7** (reference ≤4 medications)	1.28 (1.12, 1.45)	0.0003	1.21 (1.06, 1.38)	0.006	1.44 (1.06, 1.96)	0.02	1.16 (1.00, 1.34)	0.06
**8–9** (reference ≤4 medications)	1.49 (1.25, 1.77)	<0.001	1.38 (1.16, 1.64)	0.0003	1.80 (1.22, 2.66)	0.003	1.29 (1.06, 1.57)	0.01
**≥ 10** (reference ≤4 medications)	1.44 (1.22, 1.70)	<0.001	1.34 (1.13, 1.59)	0.001	1.54 (1.00, 2.37)	0.05	1.29 (1.07, 1.56)	0.01
**HIV-infection**			0.59 (0.50, 0.70)	<0.001				
**Age in 10yrs increments**			1.05 (0.98, 1.13)	0.19	1.06 (0.89, 1.26)	0.51	1.04 (0.96, 1.13)	0.32
**Female** (reference male)			0.57 (0.35, 0.92)	0.02	0.55 (0.17, 1.83)	0.33	0.57 (0.34, 0.96)	0.03
**Black** (reference white)			0.99 (0.88, 1.12)	0.87	1.02 (0.77, 1.35)	0.92	0.98 (0.86, 1.12)	0.75
**Hispanic** (reference white)			0.78 (0.63, 0.96)	0.02	0.88 (0.55, 1.41)	0.60	0.74 (0.59, 0.94)	0.01
**Current smoker** (reference never)			0.95 (0.82, 1.09)	0.43	1.21 (0.87, 1.69)	0.26	0.89 (0.76, 1.04)	0.15
**Past smoker** (reference never)			1.06 (0.89, 1.26)	0.51	1.03 (0.68, 1.57)	0.88	1.06 (0.88, 1.28)	0.52
**Alcohol related diagnosis**			1.01 (0.86, 1.20)	0.87	0.88 (0.58, 1.35)	0.57	1.04 (0.87, 1.25)	0.69
**Drug related diagnosis**			0.95 (0.79, 1.13)	0.53	1.16 (0.79, 1.70)	0.46	0.90 (0.74, 1.10)	0.31
**VACS index score 2.0 per 5 units**			1.14 (1.12, 1.16)	<0.001	1.11 (1.07, 1.15)	<0.001	1.15 (1.13, 1.18)	<0.001

MICU–medical intensive care unit; PWH–people with HIV; PWoH–people without HIV. Harrell’s C-index for the unadjusted model was 0.53 and for adjusted 0.64 (for both unstratified and stratified models).

In adjusted models, polypharmacy conferred an increased odds of 1-year MICU admission, adjusted odds ratio (aOR) (95% CI) = 1.28 (1.14, 1.43) (S2 Table). Severity of illness was also associated with increased odds of MICU admission. For each 5-point increase in the VACS index 2.0 score, there was a 14% increase in the odds of 1-year MICU admission, aOR (95% CI) = 1.14 (1.12, 1.16). HIV status, female sex, and Hispanic ethnicity were inversely associated with MICU admission. In stratified analyses by HIV status, the odds of MICU admission associated with polypharmacy was aOR (95% CI) = 1.55 (1.19, 2.01) among PWH, and aOR (95% CI) = 1.22 (1.08, 1.38) among PWoH (S2 Table). A formal test for interaction between HIV status and polypharmacy was not statistically significant, aOR (95% CI) = 1.24 (0.92, 1.65), p = 0.15.

In adjusted models using categorical non-ARV polypharmacy measures, the increased odds of 1-year MICU remained, aOR (95% CI) = 1.21 (1.06, 1.38); 1.38 (1.16, 1.64); and 1.34 (1.13, 1.59), respectively; demonstrating a dose-response relationship (Table 3). In HIV stratified models, the dose-response relationship between medication count and MICU admission was observed, with a stronger association among PWH (Table 3). Among PWH, compared to ≤4 medications, those on 5–7, 8–9, and ≥10 medications were more likely to have 1-year MICU admission (aOR (95% CI) = 1.44 (1.06, 1.96), 1.80 (1.22, 2.66), and 1.54 (1.00, 2.37), respectively). Among PWoH, compared to ≤4 medications, those on 5–7, 8–9, and ≥10 medications were more likely to have 1-year MICU admission (aOR (95% CI) = 1.16 (1.00, 1.34), 1.29 (1.06, 1.57), and 1.29 (1.07, 1.56), respectively) (Table 3). A formal test for interaction between HIV status and medication count was not statistically significant (1.23 (0.87, 1.73), p = 0.24, 1.34 (0.87, 2.08), p = 0.19, 1.14 (0.71, 1.83), p = 0.58, respectively).

In sensitivity analysis using Poisson regression, adjusting for the same covariates as in the logistic model, the results were consistent. The relative risk of MICU for those with polypharmacy compared to those without was RR (95% CI) = 1.22 (1.10, 1.35), and there was an increase in risk associated with an increase in medication count. Compared to those on ≤4 medications, risk of MICU for those on 5–7, 8–9, and ≥10 medications was 1.17 (1.04, 1.32), 1.30 (1.11, 1.52), and 1.27 (1.09, 1.48), respectively.

**Fig 2:**
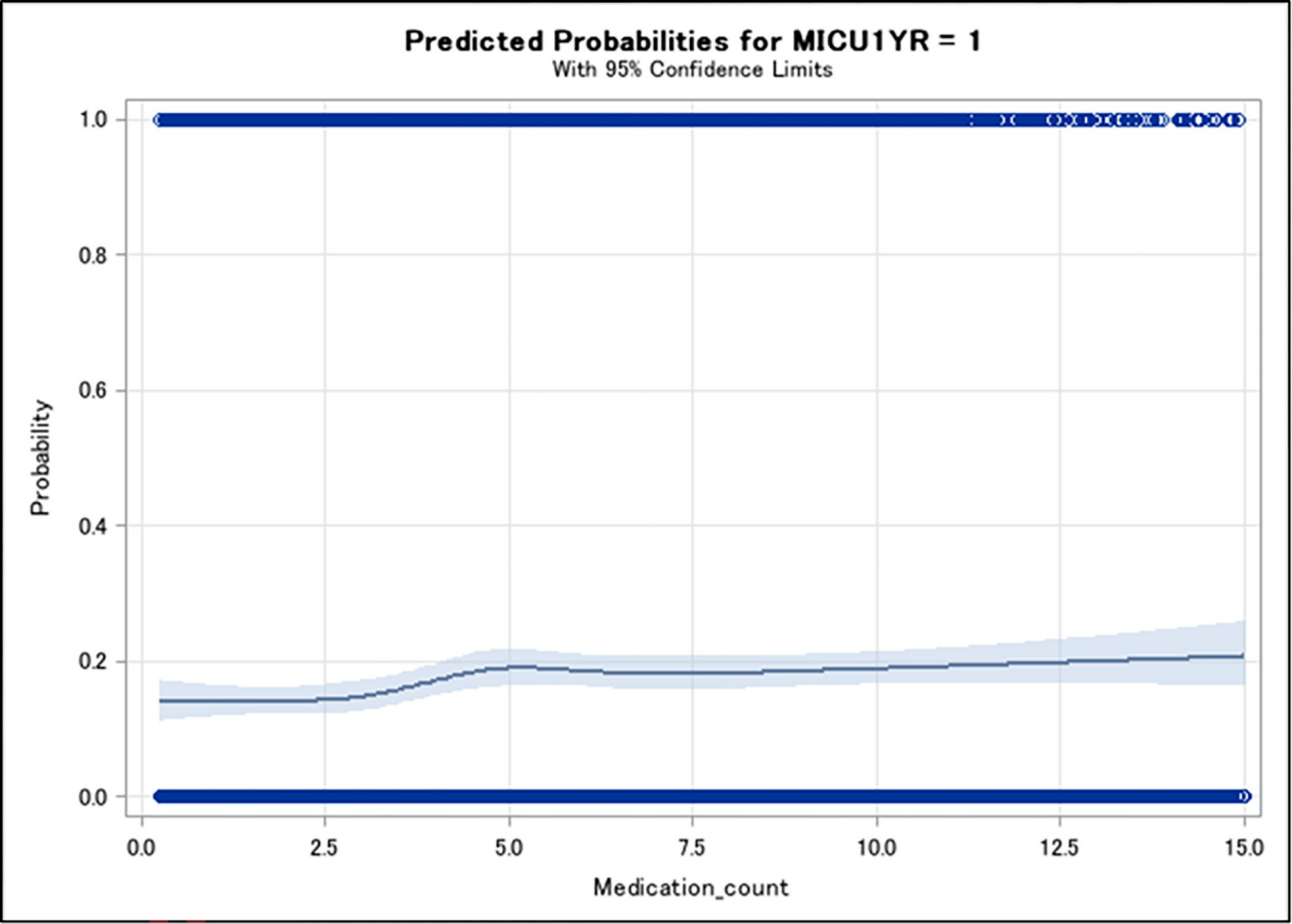
Restricted cubic spline predicted probability of 1-year MICU admission vs non-ARV medication count.

The restricted cubic spine regression results showed there was a dose response relationship between medication count and MICU admission, that our medication count categorization was reasonable and suggested an added category of <3 (Table 4 and Fig 2). In adjusted models, compared to those on <3 medications, those on 3–4, 5–7, 8–9, and ≥10 medications were more likely to have 1-year MICU admission (aOR (95% CI) = 1.26 (1.07, 1.49), 1.36 (1.16, 1.60), 1.56 (1.28, 1.89), and 151 (1.25, 1.83), respectively).

**Table 4:** Logistic regression models looking at increments of medication based on restricted cubic spline regression and MICU admission.

	Unadjusted, n = 9898		Unadjusted, n = 9898		PWH, n = 1811		PWoH, n = 8087	
	OR (95% CI)	p value	OR (95% CI)	p value	OR (95% CI)	p value	OR (95% CI)	p value
**3–4** (reference < 3 meds)	1.31 (1.11, 1.55)	0.001	1.26 (1.07, 1.49)	0.006	1.23 (0.83, 1.81)	0.30	1.27 (1.05, 1.53)	0.01
**5–7** (reference < 3 meds)	1.46 (1.25, 1.71)	<0.001	1.36 (1.16, 1.60)	0.0002	1.60 (1.11, 2.30)	0.01	1.31 (1.09, 1.56)	0.003
**8–9** (reference < 3 meds)	1.71 (1.41, 2.07)	<0.001	1.56 (1.28, 1.89)	<0.001	2.00 (1.29, 3.10)	0.002	1.46 (1.17, 1.82)	0.0007
**≥ 10** (reference < 3 meds)	1.65 (1.37, 2.00)	<0.001	1.51 (1.25, 1.83)	<0.001	1.71 (1.06, 2.75)	0.03	1.46 (1.18, 1.81)	0.0004
**HIV-infection**			0.59 (0.50, 0.70)	<0.001				
**Age in 10yrs increments**			1.04 (0.97, 1.12)	0.24	1.05 (0.89, 1.25)	0.56	1.04 (0.96, 1.12)	0.38
**Female** (reference male)			0.57 (0.35, 0.92)	0.02	0.56 (0.17, 1.86)	0.34	0.57 (0.34, 0.96)	0.03
**Black** (reference white)			0.99 (0.88, 1.12)	0.92	1.01 (0.77, 1.34)	0.92	0.98 (0.86, 1.12)	0.81
**Hispanic** (reference white)			0.78 (0.63, 0.97)	0.02	0.88 (0.55, 1.41)	0.60	0.75 (0.59, 0.95)	0.02
**Current smoker** (reference never)			0.95 (0.82, 1.09)	0.43	1.21 (0.87, 1.68)	0.27	0.89 (0.77, 1.04)	0.15
**Past smoker** (reference never)			1.06 (0.89, 1.25)	0.52	1.03 (0.67, 1.56)	0.91	1.06 (0.88, 1.28)	0.52
**Alcohol related diagnosis**			1.02 (0.86, 1.20)	0.86	0.88 (0.58, 1.35)	0.57	1.04 (0.87, 1.25)	0.69
**Drug related diagnosis**			0.95 (0.79, 1.13)	0.54	1.16 (0.79, 1.70)	0.46	0.90 (0.74, 1.10)	0.32
**VACS index score 2.0 per 5 units**			1.14 (1.12, 1.16)	<0.001	1.11 (1.07, 1.15)	<0.001	1.15 (1.13, 1.18)	<0.001

MICU–medical intensive care unit; PWH–people with HIV; PWoH–people without. Harrell’s C-index for the unadjusted model was 0.55 and for adjusted 0.64 (for both unstratified and stratified models)

### Polypharmacy and 10-year all-cause mortality

Among patients surviving the index hospitalization, both polypharmacy and medication count were associated with 10-year all-cause mortality (Table 5 and S3 Table). Moreover, there was a clear dose-response with incremental increase in medication count. In adjusted models, compared to those on ≤4 medications, the risk of mortality for those on 5–7, 8–9, and ≥10 medications were 18%, 28%, and 44%, respectively (Table 5). Other factors associated with 10-year mortality were MICU admission, age, smoking, and severity of illness. Of note, after adjustment for severity of illness and polypharmacy, HIV-infection, female sex, non-white, and drug related diagnosis were inversely associated with 10-year mortality (Table 5). The association of polypharmacy and mortality remained true in stratified models, with increasing mortality risk with increased medication count category (Table 5). For example, among PWH, the risk of 10-year all-cause mortality for patients on 5–7 medications (referent ≤4) was hazard ratio (HR) (95% CI) = 1.18 (1.01, 1.38); 8–9 medications, HR (95% CI) = 1.24 (1.00, 1.52); and ≥10 medications, HR (95% CI) = 1.27 (1.01, 1.59).

**Table 5:** Cox regression models looking at increments of medication count and 10-year all-cause mortality.

	Unadjusted, n = 9898		Adjusted, n = 9898		PWH, n = 1811		PWoH, n = 8087	
	HR (95% CI)	p value	HR (95% CI)	p value	HR (95% CI)	p value	HR (95% CI)	p value
**5–7** (reference ≤4 medications)	1.29 (1.21, 1.39)	<0.001	1.18 (1.10, 1.26)	<0.001	1.18 (1.01, 1.38)	0.04	1.17 (1.09, 1.27)	<0.001
**8–9** (reference ≤4 medications)	1.45 (1.33, 1.59)	<0.001	1.28 (1.16, 1.40)	<0.001	1.24 (1.00, 1.52)	0.05	1.28 (1.16, 1.42)	<0.001
**≥ 10** (reference ≤4 medications)	1.58 (1.45, 1.72)	<0.001	1.44 (1.32, 1.57)	<0.001	1.27 (1.01, 1.59)	0.04	1.48 (1.34, 1.62)	<0.001
**MICU admission**			1.80 (1.67, 1.93)	<0.001	2.04 (1.74, 2.39)	<0.001	1.74 (1.61, 1.88)	<0.001
**HIV-infection**			0.52 (0.48, 0.57)	<0.001				
**Age in 10yrs increments**			1.34 (1.29, 1.39)	<0.001	1.47 (1.34, 1.60)	<0.001	1.31 (1.25, 1.36)	<0.001
**Female** (reference male)			0.59 (0.45, 0.77)	0.0001	1.06 (0.60, 1.88)	0.85	0.52 (0.38, 0.71)	<0.001
**Black** (reference white)			0.81 (0.76, 0.86)	<0.001	0.79 (0.68, 0.92)	0.002	0.81 (0.75, 0.86)	<0.001
**Hispanic** (reference white)			0.84 (0.75, 0.93)	0.001	0.91 (0.73, 1.15)	0.44	0.81 (0.72, 0.91)	0.001
**Current smoker** (reference never)			1.38 (1.28, 1.49)	<0.001	1.67 (1.40, 1.98)	<0.001	1.32 (1.21, 1.43)	<0.001
**Past smoker** (reference never)			1.11 (1.01, 1.21)	0.03	1.13 (0.91, 1.40)	0.29	1.11 (1.00, 1.23)	0.04
**Alcohol related diagnosis**			1.06 (0.97, 1.16)	0.19	1.07 (0.85, 1.35)	0.58	1.06 (0.97, 1.17)	0.22
**Drug related diagnosis**			0.88 (0.80, 0.97)	0.01	0.87 (0.69, 1.09)	0.21	0.88 (0.80, 0.98)	0.02
**VACS index score 2.0 per 5 units**			1.20 (1.19, 1.22)	<0.001	1.18 (1.16, 1.20)	<0.001	1.21 (1.20, 1.23)	<0.001

MICU–medical intensive care unit; PWH–people with HIV; PWoH–people without

## Discussion

In a national sample of hospitalized PWH and demographically similar PWoH, polypharmacy was associated with 1-year MICU admission and 10-year mortality after controlling for demographics, HIV-infection, substance use, and severity of illness as estimated by VACS index 2.0 score. Moreover, there was a dose-response relationship observed, with increased medication count associated with increased MICU admission and mortality risk; we observed a slight drop off for MICU admission, possibly due to small sample size in hyper-polypharmacy (i.e., ≥10).

Polypharmacy is increasingly prevalent amongst PWH and PWoH as they age with multiple chronic conditions. Using the same cutoffs for non-antiretroviral medication count used in the current analysis, the Multicenter AIDS Cohort Study (MACS) identified polypharmacy in 36% and 30% of patients with and without HIV, respectively [20]. In a large cross-sectional analysis of 22,945 PWH compared with 6.6 million uninfected persons in Spain, polypharmacy was present in 33% vs. 22%, respectively, with PWH having 73% increased risk for polypharmacy [21]. These findings were consistent across all age strata except amongst those age 75 years and older. Another cohort of PWH age 60 years and older from San Francisco, CA reported non-ARV polypharmacy in 74% of patients [22]. In the current study, we identified non-ARV polypharmacy in 46% of PWH and 50% of PWoH among those without a MICU admission (mean age = 58 years). While non-ARV polypharmacy was higher amongst patients with a MICU admission, it did not differ by HIV status (57% for both groups). However, it is important to consider that PWH in this study were also on an average of 3 antiretroviral medications in addition to the non-ARV medications.

This work expands on prior work that found non-ARV medication polypharmacy was associated with hospitalization and mortality [5], and that polypharmacy associations with adverse outcomes were independent of underlying medical conditions (i.e., it was not simply sicker patients take more medications) [5, 23]. Our results suggest similar associations with increasing non-ARV medication count and serious medical illnesses leading to MICU admission.

Substantial work to identify the mechanisms of injury from polypharmacy is needed, specifically in aging PWH. Mechanisms by which polypharmacy-related harms may occur include mitochondrial toxicity and dysfunction, hepatotoxicity and liver injury, immune suppression, delirium, and drug-drug interactions [24, 25]. These combined insults could reduce resilience leading to greater risk of, and potentially more serious, critical illness and many of these may be differential by HIV status. For example, drug-drug interactions, controlling for number of medications, are more frequent amongst PWH compared with PWoH [21, 22] and are associated with significant increases in healthcare costs for PWH [26]. In addition, among PWH on ARVs admitted to the MICU, drug-drug interactions are common, with potential interactions identified for more than two-thirds of patients [27]. This highlights the importance of reducing polypharmacy, especially among person aging with HIV on ARVs.

In stratified models by HIV status, the association between polypharmacy and these adverse outcomes appeared to be stronger in PWH, although a formal test for interaction with HIV status was not significant. In the stratified models predicting 1-year MICU admission, PWH demonstrated increased risk associated with non-ARV polypharmacy compared to PWoH at every level of polypharmacy. In stratified models for mortality, PWH exhibited increased risk compared to PWoH associated with advancing age, current smoking, and MICU admission itself, suggesting increased susceptibility to harm from these physiologic insults as well, i.e., excess physiologic frailty. This was also suggested by the substantially higher VACS Index 2.0 scores among PWH compared to PWoH. Harms of polypharmacy are especially important to characterize and mitigate among aging PWH who require multiple chronic daily medications to achieve sustained control of the HIV virus and comorbid conditions.

Other potentially modifiable risk factors for mortality included smoking and MICU admission. Smoking was a particularly important risk factor for PWH, in whom smoking conferred increased risk of mortality of 67%. Based on the results from the stratified models, posterior interaction terms were tested between polypharmacy/medication count and smoking, alcohol diagnosis, and drug diagnosis in both outcomes and none were significant.

There was also a 2-fold association between MICU admission and 10-year mortality, independent of severity of illness which warrants further investigation regarding contributions of conditions such as post-intensive care syndrome on longer-term outcomes.

We note that HIV is associated with reduced odds for poor outcomes in the adjusted model, suggesting that after adjusting for severity of illness, those with HIV actually do better on average. This may be related to HIV as a means of promoting early enrollment and preventive health measure. The inverse association with race is likely due to minority veterans being, on average, less sick than minorities in the general population, due to service-connected lifetime health benefits [28]. When the VACS index was excluded from the models the relationship was no longer inverse or significant (S4 and S5 Tables).

This study has several limitations. Medication count was based on outpatient prescription fill in the VA and does not include inpatient medications, prescriptions outside the VA and Medicare, over the counter, or complementary and alternative medications. However, we did require patients to have a least one medication fill during baseline to ensure we were capturing non-ARV medication count and polypharmacy profile as completely as possible, albeit a conservative estimate. Nonetheless, this may not be generalizable to other hospitalized populations. While we determined polypharmacy based on data that was a decade ago, we were able to show that the same medications are in widespread use in more recent data (See Fig 1). In addition, this allowed us to demonstrate an association with long term mortality among MICU survivors. Moreover, Ware and colleagues looked at polypharmacy over time and found more than 50% of their sample had polypharmacy, and more than half of those had sustained or slowly increasing polypharmacy [20]. Also, while PWH in our sample were demographically similar to PWoH comparators, the strict 1:2 matching employed in the VACS cohort was not preserved after the exclusions required for this analysis. Further, hospitalization and MICU admissions are increasingly related to non-HIV related chronic comorbid conditions among PWH, and polypharmacy may only become of greater impact on health outcomes for aging PWH [29, 30]. Women were not adequately represented in our sample. Future work examining polypharmacy in cohorts with more representation from women would be important to further examine sex differences with regards to polypharmacy and health outcomes like MICU admission and 10-year mortality. Finally, Harrell’s C-index (c-stats) was low to moderate which indicate model precision was moderate. We did not have hospital-related variables in this dataset which could add to model precision.

An important strength of our analysis is that we looked at both polypharmacy and incremental categories of medication count, showing there is a dose-response relationship with outcomes. Further, we adjusted for important factors, such as age, substance use, and severity of illness. Finally, we were able to demonstrate the long-lasting impact of polypharmacy and long-term outcomes in PWH and PWoH.

## Conclusions

Our analysis suggests that polypharmacy is associated with increase odds of 1-year MICU admissions and 10-year all-cause mortality risk, and this association persisted after adjusting for demographics, substance use and severity of illness. Increasing counts of non-antiretroviral medication monotonically increased risk. Further research is needed to determine if medication de-prescribing and reconciliation can reduce the risk of MICU admission without impacting quality of life or survival.

## Supporting information

S1 Table ICD-9 codes for alcohol and drug related diagnosis. (DOCX)

S2 Table Logistic regression models looking at polypharmacy and MICU admission. (DOCX)

S3 Table Cox regression models looking at polypharmacy and 10-year all-cause mortality. (DOCX)

S4 Table Logistic regression models excluding severity of illness (i.e., VACS index score). (DOCX)

S5 Table Cox regression models excluding severity of illness (i.e., VACS index score). (DOCX)

S1 Fig Conceptual model of the study period of the entire dataset. (DOCX)
