# Supplementary material for: Polypharmacy and medical intensive care unit (MICU) admission and 10-year all-cause mortality risk among hospitalized patients with and without HIV
Source: PLoS One. 2022 Oct 27;17(10):e0276769. doi: 10.1371/journal.pone.0276769 (PMC9612570; doi:10.1371/journal.pone.0276769)
Supplement: S1 Table — (DOCX) [file pone.0276769.s001.docx]

**Table S1. ICD-9 codes for alcohol and drug related diagnosis**

| **Alcohol related** | | **Drug related** | | | |
| --- | --- | --- | --- | --- | --- |
| 291 | 790.3 | 292 | 304.43 | 305.22 | 305.9 |
| 291 | 980 | 292 | 304.5 | 305.23 | 305.9 |
| 291.1 | 980.8 | 292.1 | 304.51 | 305.29 | 305.91 |
| 291.2x | 980.9 | 292.11 | 304.52 | 305.3 | 305.92 |
| 291.3 | E860.0 | 292.12 | 304.53 | 305.31 | 305.93 |
| 291.4 | E860.1 | 292.2 | 304.6 | 305.32 |  |
| 291.5 | E860.8 | 304 | 304.6 | 305.33 |  |
| 291.8 | E860.9 | 304 | 304.61 | 305.4 |  |
| 291.81 | 357.5 | 304.01 | 304.62 | 305.41 |  |
| 291.89 | 425.5 | 304.02 | 304.63 | 305.42 |  |
| 291.9 | 535.3 | 304.03 | 304.7 | 305.43 |  |
| 303 | 571 | 304.09 | 304.7 | 305.5 |  |
| 303 | 571.1 | 304.1 | 304.71 | 305.51 |  |
| 303 | 571.2 | 304.11 | 304.72 | 305.52 |  |
| 303.01 | 571.3 | 304.12 | 304.73 | 305.53 |  |
| 303.02 | V11.3 | 304.13 | 304.8 | 305.6 |  |
| 303.03 |  | 304.2 | 304.8 | 305.61 |  |
| 303.9 |  | 304.21 | 304.81 | 305.62 |  |
| 303.9 |  | 304.22 | 304.82 | 305.63 |  |
| 303.91 |  | 304.23 | 304.83 | 305.7 |  |
| 303.92 |  | 304.3 | 304.9 | 305.71 |  |
| 303.93 |  | 304.31 | 304.9 | 305.72 |  |
| 305 |  | 304.32 | 304.91 | 305.73 |  |
| 305 |  | 304.33 | 304.92 | 305.8 |  |
| 305.01 |  | 304.4 | 304.93 | 305.81 |  |
| 305.02 |  | 304.41 | 305.2 | 305.82 |  |
| 305.03 |  | 304.42 | 305.21 | 305.83 |  |
