# Supplementary material for: Polypharmacy and medical intensive care unit (MICU) admission and 10-year all-cause mortality risk among hospitalized patients with and without HIV
Source: PLoS One. 2022 Oct 27;17(10):e0276769. doi: 10.1371/journal.pone.0276769 (PMC9612570; doi:10.1371/journal.pone.0276769)
Supplement: S2 Table — (DOCX) [file pone.0276769.s002.docx]

**Table S2. Logistic regression models looking at polypharmacy and MICU admission**

|  | **Unadjusted,** n=9898 | | **Adjusted,** n=9898 | | **PWH,** n=1811 | | **PWoH,** n=8087 | |
| --- | --- | --- | --- | --- | --- | --- | --- | --- |
|  | **OR**  **(95% CI)** | **p value** | **OR**  **(95% CI)** | **p value** | **OR**  **(95% CI)** | **p value** | **OR**  **(95% CI)** | **p value** |
| **Polypharmacy**  **(5 or more medications)** | 1.36  (1.22, 1.52) | <0.001 | 1.28  (1.14, 1.43) | <0.001 | 1.55  (1.19, 2.01) | 0.001 | 1.22  (1.08, 1.38) | 0.002 |
| **HIV-infection** |  |  | 0.59  (0.50, 0.70) | <0.001 |  |  |  |  |
| **Age in 10yrs increments** |  |  | 1.05  (0.98, 1.13) | 0.17 | 1.06  (0.89, 1.26) | 0.51 | 1.04  (0.96, 1.13) | 0.30 |
| **Female** (reference male) |  |  | 0.57  (0.35, 0.92) | 0.02 | 0.55  (0.16, 1.82) | 0.33 | 0.57  (0.34, 0.95) | 0.03 |
| **Black** (reference white) |  |  | 0.99  (0.88, 1.11) | 0.83 | 1.02  (0.77, 1.35) | 0.92 | 0.98  (0.86, 1.11) | 0.72 |
| **Hispanic** (reference white) |  |  | 0.77  (0.63, 0.96) | 0.02 | 0.88  (0.55, 1.40) | 0.58 | 0.74  (0.58, 0.94) | 0.01 |
| **Current smoker** (reference never) |  |  | 0.95  (0.82, 1.09) | 0.43 | 1.20  (0.86, 1.68) | 0.28 | 0.89  (0.76, 1.04) | 0.14 |
| **Past smoker** (reference never) |  |  | 1.06  (0.90, 1.26) | 0.49 | 1.03  (0.68, 1.57) | 0.89 | 1.07  (0.89, 1.29) | 0.50 |
| **Alcohol related diagnosis** |  |  | 1.01  (0.86, 1.20) | 0.89 | 0.88  (0.58, 1.35) | 0.57 | 1.04  (0.86, 1.24) | 0.71 |
| **Drug related diagnosis** |  |  | 0.95  (0.79, 1.13) | 0.54 | 1.15  (0.78, 1.70) | 0.47 | 0.90  (0.74, 1.10) | 0.32 |
| **VACS index score 2.0 per 5 units** |  |  | 1.14  (1.12, 1.16) | <0.001 | 1.11  (1.07, 1.15) | <0.001 | 1.15  (1.13, 1.18) | <0.001 |

MICU – medical intensive care unit; Polypharmacy defined as receipt of ≥ 5 chronic outpatient medications; PWH – people with HIV; PWoH – people without HIV. Harrell's C-index for the unadjusted model was 0.54, for adjusted 0.63, and 0.64 for both PWH and PWoH models.
