# Supplementary material for: Polypharmacy and medical intensive care unit (MICU) admission and 10-year all-cause mortality risk among hospitalized patients with and without HIV
Source: PLoS One. 2022 Oct 27;17(10):e0276769. doi: 10.1371/journal.pone.0276769 (PMC9612570; doi:10.1371/journal.pone.0276769)
Supplement: S3 Table — (DOCX) [file pone.0276769.s003.docx]

**Table S3. Cox regression models looking at polypharmacy and 10-year** **all-cause mortality**

|  | **Unadjusted,** n=9898 | | **Adjusted,** n=9898 | | **PWH,** n=1811 | | **PWoH,** n=8087 | |
| --- | --- | --- | --- | --- | --- | --- | --- | --- |
|  | **HR**  **(95% CI)** | **p value** | **HR**  **(95% CI)** | **p value** | **HR**  **(95% CI)** | **p value** | **HR**  **(95% CI)** | **p value** |
| **Polypharmacy (5 or more medications)** | 1.40  (1.32, 1.48) | <0.001 | 1.26  (1.19, 1.34) | <0.001 | 1.21  (1.06, 1.39) | 0.01 | 1.27  (1.19, 1.36) | <0.001 |
| **MICU admission** |  |  | 1.80  (1.68, 1.93) | <0.001 | 2.04  (1.74, 2.39) | <0.001 | 1.74  (1.61, 1.88) | <0.001 |
| **HIV-infection** |  |  | 0.52  (0.48, 0.57) | <0.001 |  |  |  |  |
| **Age in 10yrs increments** |  |  | 1.33  (1.29, 1.38) | <0.001 | 1.47  (1.34, 1.60) | <0.001 | 1.31  (1.25, 1.36) | <0.001 |
| **Female** (reference male) |  |  | 0.58  (0.44, 0.77) | 0.0001 | 1.06  (0.59, 1.87) | 0.86 | 0.51  (0.37, 0.70) | <0.001 |
| **Black** (reference white) |  |  | 0.80  (0.76, 0.86) | <0.001 | 0.79  (0.68, 0.91) | 0.002 | 0.80  (0.75, 0.86) | <0.001 |
| **Hispanic** (reference white) |  |  | 0.83  (0.74, 0.92) | 0.0004 | 0.91  (0.72, 1.14) | 0.42 | 0.80  (0.71, 0.91) | 0.0003 |
| **Current smoker** (reference never) |  |  | 1.37  (1.27, 1.48) | <0.001 | 1.66  (1.40, 1.98) | <0.001 | 1.31  (1.21, 1.43) | <0.001 |
| **Past smoker** (reference never) |  |  | 1.11  (1.02, 1.22) | 0.02 | 1.13  (0.91, 1.40) | 0.27 | 1.11  (1.01, 1.23) | 0.04 |
| **Alcohol related diagnosis** |  |  | 1.06  (0.97, 1.15) | 0.23 | 1.07  (0.85, 1.35) | 0.59 | 1.06  (0.96, 1.16) | 0.27 |
| **Drug abuse and dependence** |  |  | 0.88  (0.80, 0.97) | 0.01 | 0.87  (0.69, 1.08) | 0.21 | 0.89  (0.80, 0.99) | 0.03 |
| **VACS index score 2.0 per 5 units** |  |  | 1.20  (1.19, 1.22) | <0.001 | 1.18  (1.16, 1.20) | <0.001 | 1.21  (1.20, 1.23) | <0.001 |

Polypharmacy defined as receipt of ≥ 5 chronic outpatient medications; MICU – medical intensive care unit; PWH – people with HIV; PWoH – people without HIV
