# Supplementary material for: Polypharmacy and medical intensive care unit (MICU) admission and 10-year all-cause mortality risk among hospitalized patients with and without HIV
Source: PLoS One. 2022 Oct 27;17(10):e0276769. doi: 10.1371/journal.pone.0276769 (PMC9612570; doi:10.1371/journal.pone.0276769)
Supplement: S4 Table — (DOCX) [file pone.0276769.s004.docx]

**Table S4. Logistic regression models excluding severity of illness (i.e., VACS index score)**

|  | **MICU admission** | | **Mortality** | |
| --- | --- | --- | --- | --- |
|  | **Adjusted,** n=9898 | | **Adjusted,** n=9898 | |
|  | **OR (95% CI)** | **p value** | **HR (95% CI)** | **p value** |
| **Polypharmacy (5 or more medications)** | 1.31 (1.17, 1.46) | <0.001 | 1.28 (1.20, 1.35) | <0.001 |
| **MICU admission** |  |  | 2.09 (1.95, 2.24) | <0.001 |
| **HIV-infection** | 1.03 (0.90, 1.19) | 0.65 | 1.14 (1.06, 1.23) | 0.0006 |
| **Age in 10yrs increments** | 1.30 (1.22, 1.39) | <0.001 | 1.73 (1.68, 1.80) | <0.001 |
| **Female** (reference male) | 0.56 (0.35, 0.91) | 0.02 | 0.58 (0.44, 0.76) | <0.001 |
| **Black** (reference white) | 1.09 (0.97, 1.23) | 0.15 | 0.95 (0.89, 1.01) | 0.08 |
| **Hispanic** (reference white) | 0.80 (0.65, 0.99) | 0.04 | 0.89 (0.80, 0.99) | 0.04 |
| **Current smoker** (reference never) | 1.02 (0.89, 1.17) | 0.77 | 1.50 (1.39, 1.62) | <0.001 |
| **Past smoker** (reference never) | 1.09 (0.92, 1.29) | 0.33 | 1.13 (1.03, 1.24) | 0.01 |
| **Alcohol related diagnosis** | 1.09 (0.93, 1.29) | 0.29 | 1.15 (1.05, 1.26) | 0.002 |
| **Drug related diagnosis** | 0.93 (0.78, 1.11) | 0.39 | 0.84 (0.76, 0.93) | 0.001 |

MICU – medical intensive care unit. Harrell's C-index for the adjusted logistic models was 0.59
