# Supplementary figures and images for: Polypharmacy and medical intensive care unit (MICU) admission and 10-year all-cause mortality risk among hospitalized patients with and without HIV
Source: PLoS One. 2022 Oct 27;17(10):e0276769. doi: 10.1371/journal.pone.0276769 (PMC9612570; doi:10.1371/journal.pone.0276769)

**Figure S1. Conceptual model of the study period of the entire dataset**

**
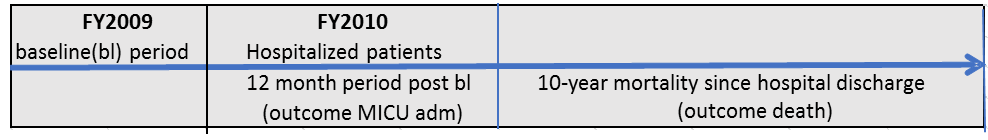
**adm = admission

Supplement: S1 Fig — (DOCX) [file pone.0276769.s006.docx]
